# Spinopelvic alignment and pubic symphysis widening are associated with postpartum low back pain: a retrospective case–control study

**DOI:** 10.3389/fbioe.2026.1804174

**Published:** 2026-04-29

**Authors:** Zhendong Huang, Zibin Zhong, Yu Min, Shun Li, Ji Li, Chengxian Song

**Affiliations:** 1 Department of Rehabilitation Medicine, The Affiliated Panyu Central Hospital, Guangzhou Medical University, Guangzhou, Guangdong, China; 2 Department of Rehabilitation Medicine, The Third Affiliated Hospital of Guangzhou Medical University, Guangzhou, Guangdong, China; 3 Guangdong Second Traditional Chinese Medicine Hospital, Guangzhou, Guangdong, China; 4 College of Bioscience and Biotechnology, Hunan Agricultural University, Changsha, Hunan, China; 5 Institute of Gerontology, Geriatric rehabilitation center, Guangzhou Geriatric Hospital, Guangzhou Medical University, Guangzhou, Guangdong, China

**Keywords:** lumbar cobb angle, lumbar lordosis angle, postpartum low back pain, pubic symphysis width, spinopelvic alignment

## Abstract

**Background:**

Postpartum low back pain (PLBP) is a frequent but often under-recognized condition that may be related to pregnancy- and delivery-induced biomechanical alterations of the lumbopelvic complex. However, imaging-based evidence linking postpartum lumbopelvic morphology to PLBP remains limited.

**Methods:**

This retrospective case–control study analyzed postpartum women (≤1 year after delivery) treated in a rehabilitation department between January 2022 and October 2023. Participants were classified into a PLBP group (n = 84) and a non-PLBP (NPLBP) group (n = 73) based on clinical assessment following guideline-based diagnostic criteria and exclusion of alternative causes. Standard weight-bearing standing anteroposterior and lateral radiographs were used to quantify lumbopelvic parameters in the coronal (Lumbar Cobb Angle, Pelvic Obliquity, Pubic Symphysis Width), transverse (Iliac Width Ratio), and sagittal planes (Pelvic Incidence, Pelvic Tilt, Sacral Slope, Lumbar Lordosis). Group comparisons were performed, and univariable and multivariable logistic regression analyses were used to identify radiographic factors independently associated with PLBP. Model performance was evaluated using the area under the receiver operating characteristic curve (AUC) and the Hosmer–Lemeshow test.

**Results:**

Baseline characteristics (age, body mass index, postpartum time, delivery mode, gravidity, and pelvic floor dysfunction) did not differ significantly between groups. Compared with the NPLBP group, the PLBP group exhibited significantly greater lumbar Cobb angle (3.82 ± 2.95° vs. 1.85 ± 1.91°), pubic symphysis width (6.85 ± 2.58 mm vs. 5.15 ± 1.64 mm), pelvic incidence (50.58 ± 8.94° vs. 46.38 ± 9.03°), sacral slope (38.01 ± 6.10° vs. 34.34 ± 7.31°), and lumbar lordosis (55.17 ± 9.58° vs. 47.15 ± 9.08°). In multivariable analysis, lumbar Cobb angle (OR 1.31 per 1°, 95% CI 1.10–1.56), lumbar lordosis (OR 1.08 per 1°, 95% CI 1.03–1.14), and pubic symphysis width (OR 1.47 per 1 mm, 95% CI 1.20–1.80) remained independently associated with PLBP. The multivariable model showed acceptable apparent discriminatory ability (AUC 0.829) and goodness-of-fit (Hosmer–Lemeshow P = 0.608).

**Conclusion:**

Coronal lumbar asymmetry, increased lumbar lordosis, and greater pubic symphysis width were independently associated with PLBP. These radiographic features may reflect altered lumbopelvic load transfer and pelvic ring stability and could inform postpartum musculoskeletal assessment and stabilization-oriented rehabilitation strategies. Prospective longitudinal studies are warranted to clarify temporality and causality.

## 1 Introduction

Postpartum low back pain (PLBP) refers to pain experienced between the lower ribcage (12th rib) and the gluteal fold in women after childbirth, particularly around the sacroiliac joints (Bergstrom et al., 2016). The pain can present unilaterally or bilaterally and often radiates to adjacent areas such as the posterior thigh, pelvic floor, or groin, but typically without neurological deficits–a key distinction from radiculopathy (Bhardwaj and Nagandla, 2014). PLBP is a common postpartum complaint, yet it is frequently under-recognized. Reported prevalence estimates differ substantially across studies—from roughly 2% to as high as 75%—and a considerable proportion of postpartum low back pain appears to be the persistence of symptoms that began during pregnancy (Barega et al., 2025). Despite its frequency, PLBP is often dismissed as a normal aftereffect of pregnancy and left inadequately managed. In reality, persistent PLBP can significantly impair a mother’s quality of life–interfering with daily activities and infant care–and contribute to psychological challenges such as elevated stress, anxiety, and even postpartum depression (Engeset et al., 2014). This not only affects the mother’s wellbeing and bonding with her newborn, but also creates an economic and caregiving burden for families and society (Gutke et al., 2014). These impacts underscore the need for greater attention to PLBP in both research and clinical care.

Current research suggests that the etiology of PLBP is multifactorial, involving a combination of hormonal, biomechanical, and psychological factors (Clinton et al., 2017) (Wang et al., 2023). During pregnancy, increased levels of relaxin, together with estrogen and progesterone, may promote remodeling of collagen-rich connective tissues and increase the compliance of the pubic symphysis and sacroiliac complex (Daneau et al., 2021; Vollestad et al., 2012). Experimental evidence further supports a direct biological role of relaxin in the pelvic ring: relaxin receptor (RXFP1) signaling has been demonstrated in pubic symphyseal tissues, and deletion of this pathway reduces collagen turnover and pubic symphysis relaxation during pregnancy (Kaftanovskaya et al., 2015). Human data also suggest that relaxin may be more closely related to pelvic joint laxity than to pain intensity itself; serum relaxin has been associated with a positive active straight leg raise test, whereas the overall evidence for a direct association between relaxin levels and pregnancy-related pelvic girdle pain remains inconsistent (Vollestad et al., 2012; Aldabe et al., 2012). These observations are important for interpreting the radiographic parameters. Pubic symphysis width may represent the most direct imaging correlate of hormone-related pelvic ligament laxity, whereas sagittal parameters may reflect secondary adaptations in sacro-pelvic orientation and lumbosacral loading when pelvic ring stiffness is reduced (Hayden et al., 2018). Coronal and transverse parameters may be interpreted less as direct markers of hormonal change and more as indicators of compensatory asymmetry or rotational adaptation within the coupled spine-pelvis system under altered load transfer (Cruz-Medel et al., 2024). Biomechanical alterations also play a central role: the dramatic shift in a mother’s center of gravity and weight distribution during pregnancy causes an anterior tilting of the pelvis and an exaggeration of lumbar lordosis. This altered posture increases the strain on the lower back structures–tension builds in the paraspinal muscles, hip flexors, and supporting fascia–which can trigger or exacerbate pain (Popajewski et al., 2024). Indeed, the lumbar spine and pelvis function as a dynamic unit, and the orientation of the sacrum/pelvis critically influences spinal alignment and load bearing. In a neutral lumbopelvic posture, the spine’s shock-absorbing capacity is optimized and stresses are evenly distributed, preventing tissue overload (Bassani et al., 2019). Conversely, any disruption in lumbopelvic alignment may lead to uneven force distribution, tissue overuse, and instability, setting the stage for musculoskeletal pain (Chaleat-Valayer et al., 2011). In addition, psychosocial factors, such as stress and poor pain coping, may interact with these biological changes, potentially amplifying pain perception and chronicity (Aboushaar and Serrano, 2024). Overall, among the various contributors to PLBP, biomechanical changes in the lumbar–pelvic region have attracted particular attention as modifiable factors that could be targeted to alleviate or prevent postpartum pain. Notably, some studies have observed that women in the postpartum period exhibit residual morphological changes in their lumbopelvic region. For example, a persistently widened pelvic girdle and an enhanced curvature of the lumbar spine have been reported after delivery (Becker et al., 2015). These structural changes are thought to reflect the combined effects of residual pregnancy-related ligamentous laxity and the mechanical demands placed on the spine and pelvis. However, the association between such postpartum anatomical changes and the development of PLBP remains unclear. Most prior investigations into pregnancy-related back pain have relied on clinical assessments or self-reported symptoms without imaging, and thus the biomechanical link between postpartum anatomical changes and pain is not well established. To address these gaps, our study will focus on quantifying lumbar–pelvic morphological changes and assessing their associations with PLBP.

Based on pre-existing radiographs from our institutional database, we will extract lumbopelvic morphometric and alignment parameters in the coronal, transverse, and sagittal planes, and examine differences in lumbopelvic morphology between women with and without PLBP. Through this imaging-based analysis, we aim to identify lumbar–pelvic morphological changes associated with PLBP. We anticipate that these findings will deepen understanding of the biomechanical context of PLBP and provide evidence to inform more precise and effective strategies for the prevention and management of postpartum lumbopelvic pain.

## 2 Methods

### 2.1 Study population

A retrospective case-control study was conducted at the Third Affiliated Hospital of Guangzhou Medical University and was approved by the hospital’s Ethics Committee (approval number: 2024-018). This study analyzed retrospective imaging data obtained from the electronic medical record system, and no additional examinations or interventions were performed for this study. All data were collected prior to the initiation of the research, fully anonymized, and processed in accordance with the General Data Protection Regulation (GDPR) to ensure participant privacy and data security. Women who underwent lumbopelvic radiographs within 12 months after delivery in the Department of Rehabilitation Medicine between January 2022 and October 2023 were eligible. The comparison group was drawn from a contemporaneous institutional postpartum assessment dataset established independently of the present study and consisted of postpartum women from the same department and study period who had undergone lumbopelvic radiography during the postpartum period for reasons unrelated to postpartum low back pain and had no documented postpartum low back pain at the index visit; these pre-existing radiographs were secondarily analyzed only after de-identification and ethics approval. Inclusion criteria: ① Full-term parturient women aged 18–40 years, within 1 year postpartum; ② No history of low back pain or trauma prior to pregnancy; ③ With reference to the 2008 European guidelines for the diagnosis of pregnancy-related low back pain, PLBP was diagnosed after excluding other causes of low back pain based on medical history and physical examination (Vleeming et al., 2008); ④ No postpartum low back pain for the non-postpartum low back pain (NPLBP) group; ⑤ Complete radiological data. Exclusion criteria: ① Concurrent factors that may cause low back pain, such as rheumatic diseases, discogenic diseases, pregnancy-related osteoporosis, scoliosis, etc.; ② Concurrent spinal, pelvic, or intraspinal tumors, lumbar tuberculosis, or other related spinal or pelvic diseases; ③ Severe functional abnormalities in major organs such as the heart, liver, or kidneys; ④ Underweight (BMI<18.5 kg/m^2^) or obesity (BMI≥28.0 kg/m^2^), according to the Chinese adult BMI classification criteria; ⑤ Received related treatment within the past month. A total of 157 patients met the criteria, including 84 in the PLBP group and 73 in the NPLBP group.

### 2.2 Data collection and definitions

**FIGURE 1:**
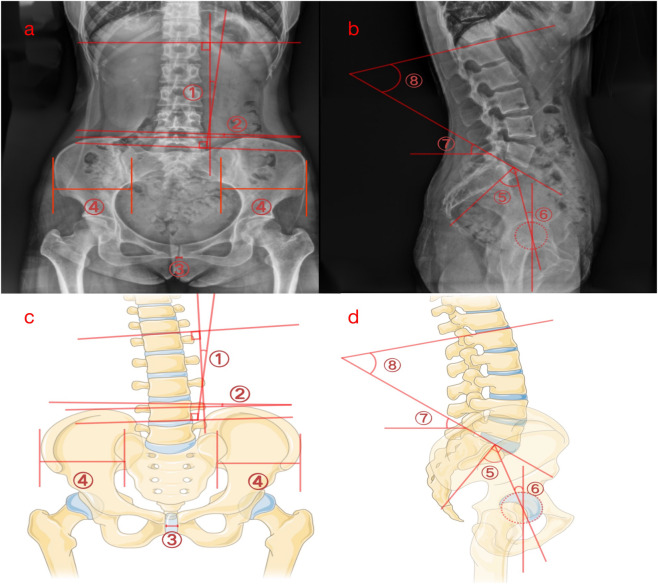
Standing anteroposterior radiograph **(a)**. Standing lateral radiograph **(b)**. Anteroposterior model diagram **(c)**. Lateral model diagram **(d)**. Lumbar Cobb Angle (①). Pelvic Obliquity (②). Pubic Symphysis Width (③). Bilateral iliac bone width (④). Pelvic Incidence angle (⑤). Pelvic Tilt Angle (⑥). Sacral Slope angle (⑦). Lumbar Lordosis Angle (⑧).

**FIGURE 2:**
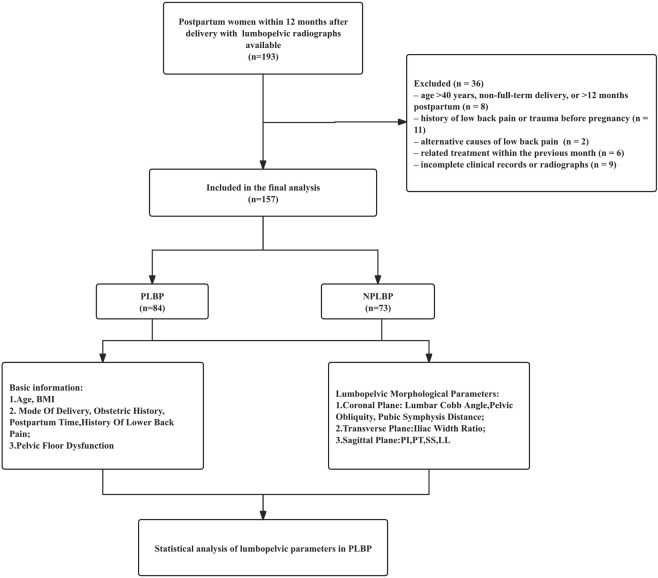
Experimental flowchart.

Basic information was collected from the medical records, including age, BMI, mode of delivery, obstetric history, postpartum time, history of low back pain, postpartum pelvic floor dysfunction, and past medical history. BMI was calculated as body weight in kilograms divided by height in meters squared (kg/m^2^), using body weight and height recorded in the medical records. In this study, postpartum time was defined as the interval between delivery and the index radiographic examination (months). According to the medical records, low back pain in the PLBP group developed after delivery; however, the exact interval from delivery to symptom onset was not consistently documented and therefore could not be analysed. In the imaging viewing system, the X-ray films taken by the patients were retrieved and extracted. All included examinations consisted of standardized anteroposterior and lateral lumbopelvic radiographs acquired in an upright weight-bearing standing position using a digital radiography system (Carestream Health, Inc., DRX-Evolution Plus, Rochester, NY, USA). The built-in software of the viewing system was utilized to measure the radiographic parameters of the lumbar spine and pelvis in the coronal, transverse, and sagittal planes, as depicted in Figure 1. Coronal Plane Parameters: ① Lumbar Cobb Angle: Refer to the Cobb angle of the spine, and draw a horizontal line on the upper edge of the upper vertebra and the lower edge of the lower vertebra in the lumbar scoliosis segment. The angle between the vertical lines of these two lines is the Lumbar Cobb Angle. ② Pelvic Obliquity (PO): The angle between the line connecting the two iliac crests and the horizontal line. ③ Pubic Symphysis Width: Measure the distance at the widest part of the left and right pubic bone branches with a horizontal line. Transverse Plane Parameters: ④ Iliac Width Ratio: Measure the width of both iliac bones and calculate the Iliac Width Ratio (smaller/larger). Sagittal Plane Parameters: ⑤ Pelvic Incidence (PI): Draw a straight line connecting the center of the femoral head and the midpoint of the upper end plate of the sacrum, and then draw a vertical line at the midpoint of the upper end plate of the sacrum. The angle between the two lines is the Pelvic Incidence angle. ⑥ Pelvic Tilt Angle (PT): Draw a line from the center of the femoral head to the midpoint of the upper end plate of the sacrum, and draw a vertical reference line from the center of the femoral head. The angle between the line and the vertical reference line is the Pelvic Tilt Angle. ⑦ Sacral Slope (SS): Draw a tangent to the upper end plate of the sacrum and measure the angle between it and the horizontal line, which is the Sacral Slope Angle. ⑧ Lumbar Lordosis Angle (LL): Draw tangents to the upper end plate of L1 vertebra and the upper end plate of the sacrum, and the angle formed by the two end plate tangents is the Lumbar Lordosis Angle. The entire experimental process is shown in Figure 2.

### 2.3 Statistical methods

Statistical analyses were performed using Python (version 3.11.2; statsmodels 0.14.3 and scikit-learn 1.4.2). Continuous variables were summarized as mean ± standard deviation for descriptive consistency. For between-group comparisons, normality was assessed separately in each group using the Shapiro–Wilk test. If both groups were approximately normally distributed, homogeneity of variance was evaluated using Levene’s test. An independent-samples t-test was used when equal variances were satisfied, whereas Welch’s t-test was used when variances were unequal. If either group deviated from normality, the Mann–Whitney U test was applied. Categorical variables were presented as number (percentage) and compared using the χ^2^ test or Fisher’s exact test, as appropriate. As this was a retrospective case–control study, the sample size was determined by all eligible postpartum women with complete clinical and radiographic data available during the predefined study period. To address whether the available sample was sufficient to detect between-group differences in radiographic parameters, a sensitivity power analysis was additionally performed. Univariable logistic regression was used to examine the association between each variable and PLBP. Variables with a univariable P value <0.10 were retained as candidate variables for multivariable analysis; no additional covariates were forced into the multivariable model due to the limited sample size. Postpartum timing was considered a clinically important source of heterogeneity; therefore, it was additionally summarized using month-based categories (1–2, 3–6, and 7–12 months), and two sensitivity analyses were performed. Because PI, PT, and SS follow the geometric identity PI = PT + SS, at most one of these parameters was entered into the multivariable model to avoid multicollinearity. Multicollinearity among the remaining candidate variables was assessed using variance inflation factors (VIF), and VIF >5 was considered indicative of problematic collinearity. A multivariable logistic regression model was then fitted to estimate adjusted associations between radiographic variables and PLBP. For descriptive purposes, the apparent discriminatory ability of the fitted model was summarized using the area under the receiver operating characteristic curve (AUC), and model fit was assessed with the Hosmer–Lemeshow goodness-of-fit test. All tests were two-sided and P < 0.05 was considered statistically significant.

## 3 Results

### 3.1 Baseline data

This study included a total of 157 patients, with 84 in the PLBP group and 73 in the NPLBP group. There were no statistically significant differences between the two groups in terms of age, BMI, postpartum time, postpartum time category, mode of delivery, gravidity, or comorbidity with pelvic floor dysfunction (all P > 0.05; Table 1).

**TABLE 1:** Baseline characteristics and between-group analysis.

Characteristic	PLBP	NPLBP	*P*
Age, years	31.62 ± 3.77	31.53 ± 4.22	0.539
BMI, kg/m^2^	22.47 ± 2.48	22.80 ± 2.81	0.433
Postpartum time, months	4.39 ± 2.65	4.21 ± 2.86	0.370
Postpartum time category			
1–2 months	24 (28.57%)	22 (30.14%)	0.777
3–6 months	45 (53.57%)	41 (56.16%)	
7–12 months	15 (17.86%)	10 (13.70%)	
Mode of delivery			
Vaginal delivery	60 (71.43%)	54 (73.97%)	0.721
Cesarean section	24 (28.57%)	19 (26.03%)	
Gravidity			
Once	59 (70.24%)	44 (60.27%)	0.350
Twice	23 (27.38%)	25 (34.25%)	
Three times	2 (2.38%)	2 (2.74%)	
More than three times	0 (0%)	2 (2.74%)	
Comorbid with pelvic floor dysfunction			
Yes	46 (54.76%)	45 (61.64%)	0.384
No	38 (45.24%)	28 (38.36%)	

P values for age and postpartum time were obtained using the Mann–Whitney U test; P values for BMI were obtained using the independent-samples t-test; P values for postpartum time category, mode of delivery, and comorbid pelvic floor dysfunction were obtained using the χ^2^ test; and P values for gravidity were obtained using the Fisher’s exact test.

### 3.2 Sensitivity power analysis

Sensitivity power analysis using G*Power showed that, with the observed sample sizes (PLBP n = 84; NPLBP n = 73), the present study had 80% power to detect a minimum standardized mean difference of Cohen’s d = 0.451 at a two-sided α level of 0.05. Based on the pooled standard deviation of each parameter, this corresponded to minimum detectable between-group differences of 1.14° for Lumbar Cobb Angle, 4.05° for Pelvic Incidence, 3.21° for Pelvic Tilt, 3.02° for Sacral Slope, 4.22° for Lumbar Lordosis, 0.46° for Pelvic Obliquity, and 0.99 mm for Pubic Symphysis Width. The observed between-group differences in Lumbar Cobb Angle (1.97°), Pelvic Incidence (4.20°), Sacral Slope (3.67°), Lumbar Lordosis (8.02°), and Pubic Symphysis Width (1.70 mm) met or exceeded these thresholds, whereas the differences in Pelvic Tilt (0.87°), Pelvic Obliquity (0.08°), and Iliac Width Ratio (0.01) did not (Table 2).

**TABLE 2:** Sensitivity power analysis.

Parameter	Pooled SD	Cohen’s d	Minimum detectable difference
Coronal plane			
Lumbar Cobb Angle,°	2.52	0.78	1.14
Pelvic Obliquity,°	1.02	0.08	0.46
Pubic symphysis Width,mm	2.19	0.78	0.99
Transverse plane			
Iliac width ratio	0.04	0.25	0.02
Sagittal plane			
Pelvic incidence,°	8.98	0.47	4.05
Pelvic tilt Angle,°	7.11	0.12	3.21
Sacral slope Angle,°	6.69	0.55	3.02
Lumbar lordosis Angle,°	9.35	0.86	4.22

### 3.3 Lumbar-pelvic parameters

**FIGURE 3:**
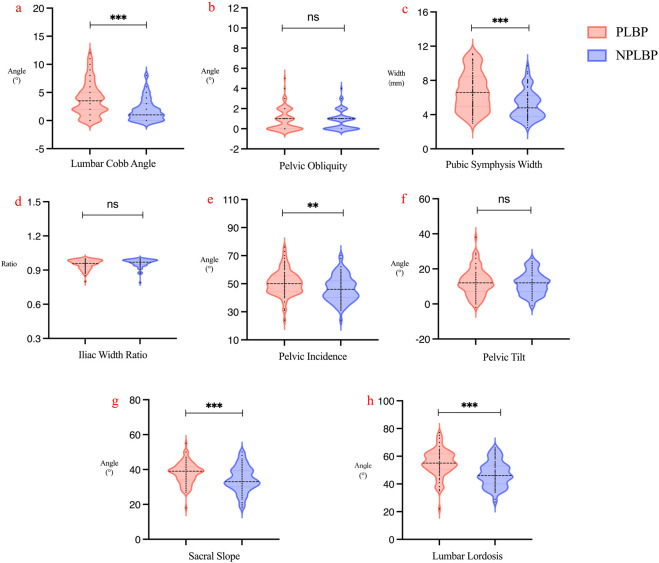
Violin plots of lumbar-pelvic parameters: **(a)** Lumbar Cobb Angle, **(b)** Pelvic Obliquity, **(c)** Pubic Symphysis Width, **(d)** Iliac Width Ratio, **(e)** Pelvic Incidence, **(f)** Pelvic Tilt, **(g)** Sacral Slope, and **(h)** Lumbar Lordosis. *p < 0.05; **p < 0.01; ***p < 0.001; ns, not significant.

In the coronal plane, there was no statistically significant difference in PO between the two groups, while the Lumbar Cobb Angle and Pubic Symphysis Width were significantly greater in the PLBP group compared to NPLBP group (P < 0.01). In the transverse plane, the iliac width ratio showed no statistically significant difference (P > 0.05). Sagittal plane assessment revealed that PI, SS, and LL were significantly higher in the PLBP group (P < 0.05), while PT showed no significant difference between the two groups (P > 0.05) (Table 3; Figure 3).

**TABLE 3:** Comparison of lumbar-pelvic parameters.

Parameter	PLBP	NPLBP	*P*
Coronal plane			
Lumbar Cobb Angle,°	3.82 ± 2.95	1.85 ± 1.91	<0.001
Pelvic Obliquity,°	0.90 ± 1.08	0.82 ± 0.93	0.833
Pubic symphysis Width,mm	6.85 ± 2.58	5.15 ± 1.64	<0.001
Transverse plane			
Iliac width ratio	0.95 ± 0.04	0.96 ± 0.04	0.120
Sagittal plane			
Pelvic Incidence,°	50.58 ± 8.94	46.38 ± 9.03	0.004
Pelvic tilt Angle,°	12.65 ± 7.64	11.78 ± 6.47	0.443
Sacral slope Angle,°	38.01 ± 6.10	34.34 ± 7.31	<0.001
Lumbar lordosis Angle,°	55.17 ± 9.58	47.15 ± 9.08	<0.001

P values for Lumbar Cobb Angle, Pelvic Obliquity, Pubic Symphysis Width, and Iliac Width Ratio were obtained using the Mann–Whitney U test; P values for Pelvic Incidence, Pelvic Tilt Angle, Sacral Slope Angle, and Lumbar Lordosis Angle were obtained using the independent-samples t-test.

### 3.4 Logistic regression analyses

**FIGURE 4:**
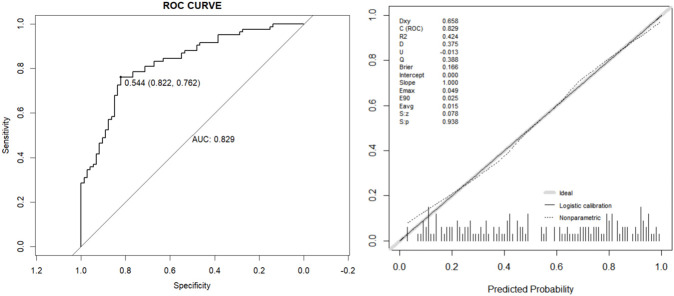
ROC curve and calibration curve.

In univariable logistic regression, Lumbar Cobb Angle (OR 1.41 per 1°, 95% CI 1.20–1.65; P < 0.001), PI (OR 1.05 per 1°, 95% CI 1.02–1.10; P = 0.005), SS (OR 1.09 per 1°, 95% CI 1.03–1.14; P = 0.001), LL (OR 1.10 per 1°, 95% CI 1.05–1.14; P < 0.001), and Pubic Symphysis Width (OR 1.50 per 1mm, 95% CI 1.25–1.81; P < 0.001) were associated with higher odds of PLBP. Variance inflation factors indicated no problematic multicollinearity among candidate variables (VIF range 1.05–1.46). In multivariable logistic regression, Lumbar Cobb Angle (OR 1.31 per 1°, 95% CI 1.10–1.56; P = 0.002), LL (OR 1.08 per 1°, 95% CI 1.02–1.15; P = 0.002), and Pubic Symphysis Width (OR 1.47 per 1mm, 95% CI 1.20–1.80; P < 0.001) remained independently associated with PLBP, whereas PI was not significant after adjustment. The model showed good discrimination (AUC 0.829, 95% CI 0.764–0.891) and no evidence of lack of fit on the Hosmer–Lemeshow test (χ^2^ = 6.35, df = 8; P = 0.608) (Figure 4; Tables 4–6).

**TABLE 4:** Univariable logistic regression analysis.

Variable	Or (95% CI)	*P*
BMI	0.95 (0.85–1.07)	0.431
Age	1.01 (0.93–1.09)	0.894
Mode of delivery	1.14 (0.56–2.30)	0.722
Gravidity	0.64 (0.38–1.10)	0.108
Pelvic floor dysfunction	0.75 (0.40–1.43)	0.384
Postpartum time	1.03 (0.91–1.15)	0.669
Lumbar Cobb angle	1.41 (1.20–1.65)	<0.001
Pelvic incidence	1.05 (1.02–1.10)	0.005
Pelvic tilt angle	1.02 (0.97–1.06)	0.441
Sacral slope angle	1.09 (1.03–1.14)	0.001
Lumbar lordosis angle	1.10 (1.05–1.14)	<0.001
Pubic symphysis width	1.50 (1.25–1.81)	<0.001
Pelvic obliquity	1.09 (0.79–1.48)	0.609
Iliac width ratio	0.52 (0.23–1.21)	0.128

**TABLE 5:** Variance Inflation Factors for variables included in the multivariable analysis.

Variable	VIF
Lumbar Cobb angle	1.09
Pelvic incidence	1.41
Lumbar lordosis angle	1.46
Pubic symphysis width	1.05

**TABLE 6:** Multivariable logistic regression analysis.

Variable	Or (95% CI)	*P*
Lumbar Cobb angle	1.31 (1.10–1.56)	0.002
Pelvic incidence	1.01 (0.96–1.07)	0.588
Lumbar lordosis angle	1.08 (1.03–1.14)	0.002
Pubic symphysis width	1.47 (1.20–1.80)	<0.001

### 3.5 Sensitivity analyses

To address potential heterogeneity across postpartum stages, two sensitivity analyses were performed (Table 7). First, the primary multivariable model was refitted with postpartum time forced into the model. In this analysis, postpartum time showed a nonsignificant trend (OR 1.13 per month, 95% CI 0.98–1.30; P = 0.088), whereas Lumbar Cobb Angle (OR 1.32 per 1°, 95% CI 1.11–1.58; P = 0.002), LL (OR 1.09 per 1°, 95% CI 1.03–1.14; P = 0.001), and Pubic Symphysis Width (OR 1.48 per 1mm, 95% CI 1.21–1.81; P < 0.001) remained independently associated with PLBP; PI remained nonsignificant (OR 1.02 per 1°, 95% CI 0.97–1.07; P = 0.496). Second, in the subgroup assessed 2–6 months postpartum (n = 124; 63 PLBP and 61 NPLBP), the associations remained materially unchanged: Lumbar Cobb Angle (OR 1.37 per 1°, 95% CI 1.13–1.66; P = 0.001), LL (OR 1.10 per 1°, 95% CI 1.04–1.17; P = 0.001), and Pubic Symphysis Width (OR 1.34 per 1mm, 95% CI 1.07–1.67; P = 0.010), while PI remained nonsignificant (OR 1.00 per 1°, 95% CI 0.94–1.06; P = 0.915).

**TABLE 7:** Sensitivity analyses.

Variable	Model A: primary model + postpartum time adjustment, OR (95% CI)	*P*	Model B: restricted to 2–6 months postpartum, OR (95% CI)	*P*
Postpartum time	1.13 (0.98–1.30)	0.088	-	-
Lumbar Cobb angle	1.32 (1.11–1.58)	0.002	1.37 (1.13–1.66)	0.001
Pelvic incidence	1.02 (0.97–1.07)	0.496	1.00 (0.94–1.06)	0.915
Lumbar lordosis angle	1.09 (1.03–1.14)	0.001	1.10 (1.04–1.17)	0.001
Pubic symphysis width	1.48 (1.21–1.81)	<0.001	1.34 (1.07–1.67)	0.010

## 4 Discussion

The present study investigated whether radiographic lumbar-pelvic morphological parameters are associated with PLBP and identified three biomechanical factors that remained independently associated with PLBP after multivariable adjustment: a larger Lumbar Cobb Angle in the coronal plane, increased LL in the sagittal plane, and a wider Pubic Symphysis. In contrast, common demographic and obstetric characteristics (age, BMI, postpartum time, mode of delivery, gravidity, and comorbidity with pelvic floor dysfunction) were not significantly different between PLBP and NPLBP groups. Taken together, these findings support the concept that, within a postpartum population with broadly comparable baseline characteristics, altered load transfer and alignment in the lumbopelvic ring—rather than demographic or obstetric variables alone—may be more closely related to PLBP occurrence and persistence.

Previous studies have demonstrated that PLBP is not caused by a single factor but rather by the interaction of multiple factors. Common associated factors include age, BMI, lumbopelvic complex pain, decreased back muscle endurance, high workload, and intense physical activity (Olsson et al., 2012a). Additionally, PLBP patients often exhibit catastrophic thinking and fear-avoidance beliefs, which manifest as overly pessimistic expectations for recovery and excessive worry about movements that might trigger pain. This avoidance behavior significantly impacts quality of life (Olsson et al., 2012b). Although previous research has explored PLBP associated factors, no consensus has been reached. This study found no significant association between BMI, age, and PLBP. In terms of delivery method, some studies have shown that women who have vaginal deliveries experience PLBP less frequently and with lower severity and shorter duration compared to those with cesarean sections (Mogren, 2007; Wang et al., 2010). According to Mukkannavar et al. (2013), differences in parity may influence PLBP prevalence, potentially due to pelvic joint changes associated with multiple pregnancies. This study also examined the relationship between delivery mode, parity, postpartum period, and PLBP but found no significant associations, consistent with the findings of Burani et al. (2023). In our study, the lack of association with BMI, delivery mode, gravidity, and postpartum time may reflect (i) true weak effects of these variables compared with biomechanical factors in this specific clinical sample; (ii) limited statistical power for small-to-moderate effects; (iii) heterogeneity in unmeasured exposures (e.g., work demands, infant-care posture, sleep deprivation, physical activity, and psychosocial factors), which are difficult to capture retrospectively. Importantly, because the primary aim of this study is not risk prediction, we emphasize the interpretation of these results as associations that highlight plausible biomechanical risk pathways rather than as tools for forecasting PLBP in individuals.

Furthermore, pelvic floor muscle damage during pregnancy may contribute to PLBP. The pelvic floor muscles, in conjunction with the lumbar, core, and gluteal muscles, maintain abdominal and pelvic stability. Damage to the pelvic floor may compromise lumbopelvic stability, contributing to PLBP symptoms (Rostaminia, 2020). Increased stress in the lumbopelvic region among PLBP patients may also place additional strain on the pelvic floor, potentially exacerbating pelvic floor dysfunction (Desgagnés et al., 2022). However, both this study and Stuge et al. (2013) found no significant difference in the incidence of pelvic floor dysfunction between postpartum women with and without PLBP. The differences between our findings and those of other studies may be attributable to the limitations of the retrospective study design. Retrospective studies are prone to selection bias and may lack control over unrecognized confounding factors, potentially impacting the interpretation of PLBP associations.

A key novel observation is that the lumbar Cobb angle was greater in the PLBP group and remained independently associated with PLBP (per 1°increase: adjusted OR 1.31). Although the magnitude of curvature did not reach the classic diagnostic threshold for scoliosis, smaller degrees of asymmetry may still be clinically meaningful in the context of postpartum biomechanics. In general populations with low back pain, static postural differences and asymmetries have been reported at group level, although relationships are complex and not necessarily deterministic (Sugavanam et al., 2025). From a mechanistic perspective, even mild coronal lumbar curvature can be interpreted as an indicator of asymmetric loading and muscle activation around the lumbar spine and pelvis. During the postpartum period, repetitive asymmetric tasks—such as unilateral infant carrying, breastfeeding postures, and prolonged trunk rotation or flexion—may amplify pre-existing asymmetries or promote functional coronal deviation as a pain-avoidance strategy (Havens et al., 2020). This could increase unilateral facet joint loading, produce asymmetric paraspinal muscle fatigue, and require compensatory pelvic adjustments to maintain balance. Such a pattern would be expected to reduce the efficiency of load transfer across the lumbopelvic complex, increasing cumulative mechanical stress and contributing to pain sensitization. Notably, because our radiographs were assessed at a single postpartum time point, we cannot determine whether the larger lumbar Cobb angle predates pregnancy, is induced by pregnancy-related adaptations, or is secondary to pain-related antalgic posture. Longitudinal imaging would be essential to clarify temporality. We did not observe a statistically significant difference in PO, suggesting that coronal pelvic change may be less sensitive than lumbar coronal curvature in distinguishing participants with PLBP from those without PLBP in this case-control study. PO is influenced by multiple upstream factors, including lower-limb alignment, leg-length discrepancy, and hip contracture, and compensatory strategies may distribute coronal imbalance across the spine, pelvis, and lower extremities rather than concentrating it in a single pelvic parameter (Hamad et al., 2022). Therefore, a normal PO does not exclude clinically relevant coronal asymmetry elsewhere, and future studies may benefit from combining lumbar Cobb angle with whole-spine coronal balance metrics.

Pubic Symphysis Width emerged as a robust independent factor associated with PLBP (per 1 mm increase: adjusted OR 1.47). This finding is biomechanically plausible because the pubic symphysis is a key anterior stabilizer of the pelvic ring (Becker et al., 2015). Pregnancy-related hormonal changes (including relaxin, estrogen, and progesterone) can alter connective tissue properties and increase joint mobility (Madugalle et al., 2023). In non-pregnant adults, the normal symphyseal width is typically 3–6 mm. During pregnancy and the perinatal period, an additional 3–5 mm of widening is generally considered physiological, and the gap may reach approximately 9 mm; by contrast, an intrapubic gap greater than 10 mm is commonly used to define overt pubic symphysis diastasis (Stolarczyk et al., 2021). Physiological perinatal widening may also take several months to regress toward baseline. Therefore, the mean value observed in our PLBP group (6.85 mm at a mean postpartum time of 4.40 months) is better interpreted as persistent sub-diastatic widening or residual postpartum adaptation rather than overt pathological diastasis at the group level. When widening becomes excessive or persists postpartum, the pelvis may behave less like a closed ring and more like a structure with reduced stiffness, which can impair force closure and load transfer across the sacroiliac joints and lumbosacral junction (Hamad et al., 2022). Disruption of the anterior pelvic ring can coexist with, or contribute to, posterior pelvic ring symptoms and functional disability (Stolarczyk et al., 2021). Even when the degree of widening does not meet formal criteria for diastasis, incremental increases may still reflect greater ligamentous laxity and micro-instability, potentially increasing mechanical demand on trunk and hip stabilizers. Moreover, symptom severity is unlikely to be determined by gap width alone; it probably reflects the combined effects of residual ligamentous laxity, local inflammation, load-transfer disturbance across the pelvic ring, and individual neuromuscular adaptation (Fukano et al., 2022). In this context, the association between pubic symphysis width and PLBP can be understood as a marker of altered pelvic ring mechanics rather than a direct surrogate for pain severity. From a rehabilitation and women’s health perspective, this result has practical implications. It supports careful clinical screening for anterior pelvic pain patterns and load-transfer dysfunction in postpartum women with larger pubic symphysis width, and it provides a structural rationale for stabilization-focused interventions, such as pelvic belts as an external force-closure adjunct and individualized lumbopelvic stabilization training.

Changes in the horizontal plane of the pelvis are characterized by rotation around the spine, commonly quantified by methods such as bilateral iliac width ratio and obturator foramen ratio (Zhang et al., 2024). No significant difference was found in the iliac width ratio, suggesting that the assessed transverse-plane pelvic asymmetry or rotation is not strongly related to PLBP in our sample. However, transverse-plane assessment using standard radiographs is inherently limited because axial rotation is difficult to quantify on 2D projections and may be confounded by positioning and concurrent sagittal or coronal alignment. Therefore, the null finding should be interpreted cautiously: pelvic rotation might still be present but inadequately captured by the selected parameter. Future research should explore alternative parameters to enhance assessment accuracy.

In the sagittal plane, PI is commonly regarded as a morphology-dominant pelvic parameter, whereas PT and SS primarily reflect pelvic orientation; geometrically, PI equals the sum of PT and SS (PI = PT + SS) (Liu et al., 2020). Although PI is often treated as relatively stable in adults, small position-related variation has been reported, likely reflecting sacroiliac joint motion or measurement variability. In our study population, PI, SS, and LL were higher in the PLBP group, whereas PT did not differ significantly. In univariable analyses, both PI and SS were associated with PLBP; however, after multivariable adjustment, only LL remained independently associated. This is biologically plausible because PI, SS, and LL are strongly interrelated: PI constrains the feasible range of SS, and SS is closely coupled to the lordotic profile (Chanplakorn et al., 2011). Therefore, once LL is included in the multivariable model, the incremental explanatory value of PI or SS may diminish because LL is closer to the mechanical interface where load, muscle demand, and symptoms manifest. In other words, LL may function as a more immediate biomechanical correlate of altered lumbosacral loading, while PI represents a more upstream morphologic predisposition that is partly expressed through LL. In pregnant women, LL typically increases. In early pregnancy, core muscles can compensate for biomechanical changes, leading to minimal change in LL; however, as fetal weight increases and abdominal morphology changes, the uterus gradually moves outward and upward from the pelvis, causing a shift in the body’s center of gravity. To maintain balance, the body adjusts posture to increase LL, allowing more of the center of gravity to act on the posterior lumbar spine, thereby better supporting the anterior load and maintaining stability (Elabd and Elabd, 2021). Previous studies have shown that LL increases in PLBP patients and is associated with the occurrence of PLBP (Betsch et al., 2015), which is consistent with our findings. As LL increases, the shear force between the L4, L5, and sacral discs rises, the disc spaces narrow, and local muscle tension increases, causing microcirculatory disturbances and the accumulation of metabolic waste such as lactic acid. This activates pain receptors, leading to pain (Elabd and Elabd, 2021). When LL becomes excessively pronounced, lumbar structures are more likely to slip forward, increasing the risk of lumbar spondylolisthesis and contributing to low back pain (Liu et al., 2023). However, PT and SS should be interpreted more cautiously because they are orientation-dependent parameters derived from a single standing radiograph rather than fixed morphologic traits. Accordingly, the higher SS observed in the PLBP group may reflect either a predisposing sagittal alignment profile or a pain-adaptive posture adopted to reduce discomfort and maintain balance. This interpretation is consistent with other low back pain literature, in which posture-related associations do not establish causality (Swain et al., 2020). Although pelvic tilt may differ between people with and without low back pain, firm conclusions regarding PT, SS, and other static lumbopelvic parameters remain limited by heterogeneity and measurement variability (Sugavanam et al., 2025). Therefore, our findings support association rather than directionality.

Regarding changes in PT values in pregnant women, previous studies show mixed findings. Some studies report a posterior pelvic tilt among postpartum women (Kouhkan et al., 2015), while others indicate that pregnancy and the postpartum period may involve anterior pelvic tilt, which has been considered an associated factor for low back pain (Zhu et al., 2014). In this study, the PT values of both groups were similar to those reported for healthy subjects in other research (Zhu et al., 2014), with no statistically significant difference between the two groups, suggesting no strong association between PT and PLBP, consistent with the findings of Betsch et al. (2015). The absence of a significant between-group difference in PT and the loss of statistical significance of SS after multivariable adjustment further support this cautious interpretation. Differences between published reports and our radiographic findings may be attributable to technique, sample characteristics, timing, and pain-related postural adaptation, reinforcing the need for standardized measurement protocols and longitudinal designs.

The three independent factors identified—lumbar coronal curvature, increased LL, and widened pubic symphysis—can be coherently integrated under a load-transfer framework. The pelvis and lumbar spine act as a coupled system: changes in pelvic ring stiffness (anteriorly via the pubic symphysis) may alter posterior pelvic mechanics and sacral orientation, while increased LL can increase shear and compressive demand at the lumbosacral junction. Coronal curvature may further redistribute loads asymmetrically across discs, facets, and paraspinal muscles. When these features co-occur, the system may have reduced capacity to absorb and distribute repetitive postpartum loads efficiently, predisposing women to persistent or activity-provoked pain. In practice, these radiographic features should not be interpreted as deterministic causes of PLBP, but rather as structural markers that may help clinicians hypothesize which mechanical pathways, such as coronal asymmetry, hyperlordosis-related loading, pelvic ring laxity are likely to be relevant for an individual’s symptoms and rehabilitation strategy.

## 5 Limitations and future directions

Several limitations should be acknowledged. First, the retrospective design limits causal inference; observed associations should be interpreted as structural markers associated with PLBP rather than evidence of causality. Second, radiographic measurements provide static morphology in a controlled posture and do not capture dynamic motor control, muscle activation, or load-transfer function during daily tasks, which are central to rehabilitation medicine. Third, we lacked pre-pregnancy baseline imaging, preventing us from determining whether altered alignment parameters were pre-existing traits or postpartum adaptations. We also lacked detailed information on postpartum exercise, pelvic floor muscle training, and other rehabilitation exposures, which may have influenced neuromuscular compensation and symptom severity. Residual confounding from these unmeasured factors cannot be excluded. Fourth, unmeasured confounders—including occupational workload, breastfeeding or infant-care posture, physical activity, and psychosocial factors—may influence both alignment and pain. Lastly, despite assessing collinearity and respecting the PI = PT + SS identity, the relatively limited sample size restricts the number of covariates that can be simultaneously modeled and may affect stability of estimates.

Future research should prioritize prospective longitudinal designs with repeated assessments from late pregnancy through multiple postpartum time points, incorporating both imaging and functional measures. It would also be valuable to explore whether thresholds or combinations of the identified radiographic factors correspond to distinct clinical subtypes of PLBP that respond differently to targeted rehabilitation.

## 6 Conclusion

In this case-control study of 157 postpartum women, a larger lumbar Cobb angle, greater lumbar lordosis, and increased pubic symphysis width were independently associated with higher odds of PLBP, whereas pelvic incidence and sacral slope—although different between groups—were not independently associated after accounting for lumbar lordosis. These findings highlight clinically relevant biomechanical structural and biomechanical features associated with PLBP that may reflect coronal asymmetry, sagittal hyperlordotic loading, and reduced anterior pelvic ring stiffness, respectively. Incorporating careful assessment of coronal and sagittal spinal alignment and pubic symphysis widening into postpartum musculoskeletal evaluation may help rehabilitation clinicians recognize vulnerable patients and prioritize stabilization-oriented, individualized interventions aimed at improving lumbopelvic load transfer and functional recovery.

## Data Availability

The datasets analyzed in this study are not publicly available due to privacy and ethical restrictions related to patient data. Requests to access the datasets should be directed to the corresponding authors and are subject to approval by the relevant institution and ethics requirements.
